# Comparison of treatment persistence, adherence, and risk of exacerbation in patients with COPD treated with single-inhaler *versus* multiple-inhaler triple therapy: A prospective observational study in China

**DOI:** 10.3389/fphar.2023.1147985

**Published:** 2023-03-21

**Authors:** Ling Lin, Cong Liu, Wei Cheng, Qing Song, Yuqin Zeng, Xin Li, Dingding Deng, Dan Liu, Yan Chen, Shan Cai, Ping Chen

**Affiliations:** ^1^ Department of Respiratory and Critical Care Medicine, The Second Xiangya Hospital, Central South University, Changsha, Hunan, China; ^2^ Research Unit of Respiratory Disease, Central South University, Changsha, Hunan, China; ^3^ Diagnosis and Treatment Center of Respiratory Disease, Central South University, Changsha, Hunan, China; ^4^ Division 4 of Occupational Diseases, Hunan Prevention and Treatment Institute for Occupational Diseases, Changsha, China; ^5^ Department of Respiratory Medicine, The First Affiliated People’s Hospital, Shaoyang College, Shaoyang, China; ^6^ Department of Respiratory, The Eighth Hospital in Changsha, Hunan, China

**Keywords:** COPD, multiple-inhaler triple therapy, single-inhaler triple therapy, treatment persistence, adherence, exacerbation

## Abstract

**Aim:** This study sought to compare treatment persistence, adherence, and risk of exacerbation among patients with COPD treated with single-inhaler triple therapy (SITT) and multiple-inhaler triple therapy (MITT) in the Chinese population.

**Methods:** This was a multicenter, prospective observational study. Patients with COPD from ten hospitals in Hunan and Guangxi provinces in China were recruited from 1 January 2020 to 31 November 2021 for the study and were followed up for one year. Treatment persistence, adherence, and exacerbation rates during the 12-month follow-up were analyzed in COPD patients treated with SITT and MITT.

**Results:** A total of 1,328 patients were enrolled for final analysis, including 535 (40.3%) patients treated with SITT and 793 (59.7%) treated with MITT. Of these patients, the mean age was 64.9 years and most patients were men. The mean CAT score was 15.2 ± 7.1, and the median (IQR) FEV1% was 54.4 (31.2). The SITT group had a higher mean CAT score, more patients with mMRC >1, and lower mean FEV1% and FEV1/FVC than the MITT patients. Moreover, the proportion of patients with ≥1 exacerbation in the previous year was higher in the SITT cohort. SITT patients had, compared to MITT patients, a higher proportion of adherence (proportion of days covered, PDC) ≥0.8 (86.5% vs. 79.8%; *p* = 0.006), higher treatment persistence [HR: 1.676 (1.356–2.071), *p* < 0.001], lower risk of moderate-to-severe exacerbation [HR: 0.729 (0.593–0.898), *p* = 0.003], and severe exacerbation [HR: 0.675 (0.515–0.875), *p* = 0.003], as well as reduced all-cause mortality risk [HR: 0.475 (0.237-0.952), *p* = 0.036] during the 12-month follow-up. Persistence was related to fewer future exacerbations and mortality than non-persistence in the SITT and MITT groups.

**Conclusion:** Patients with COPD treated with SITT showed improved treatment persistence and adherence, as well as a reduction in the risk of moderate-to-severe exacerbation, severe exacerbation, and mortality compared to patients treated with MITT in the Chinese population.

**Clinical Trial Registration**: https://www.chictr.org.cn/, identifier ChiCTR-POC-17010431.

## Introduction

Chronic obstructive pulmonary disease (COPD) is a common chronic progressive respiratory disease characterized by irreversible and continuously progressive airflow limitations and recurrent respiratory symptoms (Vogelmeier et al., 2017; Celli et al., 2022; Confalonieri et al., 2023). According to large-scale global epidemiological evidence, the global prevalence of COPD is 11.7% and is increasing each year (Adeloye et al., 2015). The prevalence in patients aged 40 years or older in China is 13.7% (Wang et al., 2018). The progression of COPD in patients can lead to more severe respiratory symptoms and a worse quality of life, thus causing a significant financial burden (GBD, 2015 Disease and Injury Incidence and Prevalence Collaborators, 2016). It has become the third leading cause of death worldwide and one of the most important global public health issues (Lozano et al., 2012). The symptoms of patients with COPD include dyspnoea, wheezing, and coughing (Vogelmeier et al., 2017), whereas exacerbation may lead to more severe respiratory symptoms, an accelerated decline in lung function, and increased mortality (Seemungal et al., 1998; Soler-Cataluña et al., 2005). Therefore, it is important to control and reduce the severity of these symptoms.

Short- and long-acting bronchodilators are currently internationally recommended for relieving symptoms of COPD and slowing its progression. Bronchodilators include anticholinergic short-acting muscarinic antagonists (SAMAs), short-acting beta-2 agonists (SABAs), long-acting muscarinic antagonists (LAMAs), and long-acting beta-2 agonists (LABAs). Patients with frequent exacerbations or high blood eosinophils on the basis of receiving bronchodilators can additionally be treated with ICS (Halpin et al., 2021a). Multiple clinical trials demonstrate that triple-inhaler therapy (ICS/LABA/LAMA) has a higher impact in reducing the risk of all-cause mortality and moderate-to-severe exacerbation than ICS/LABA (Singh et al., 2016; Vestbo et al., 2017) and LABA/LAMA (Petite et al., 2018; Lipson et al., 2020). Currently, triple therapy includes single-inhaler triple therapy (SITT) with a single device and multiple-inhaler triple therapy (MITT) with two or more devices. The former mainly includes BUD/GLY/FOR (Budesonide/Glycopyrronium Bromide/Formoterol Fumarate) and FF/UMEC/VI (Fluticasone Furoate/Umeclidinium Bromide/Vilanterol). A retrospective study in the USA showed that treatment adherence and persistence in patients with SITT were better than those with MITT (Mannino et al., 2022). Several studies have compared short-term symptomatic improvement and risk of exacerbation in two cohorts. A randomized controlled study by Bremner et al. (2018) indicated no statistically significant difference in the improvement of lung function and health status after 6 months between SITT and MITT groups. However, a real-world observational study in the United Kingdom showed that patients with SITT had improved lung function and a higher proportion of CAT improvement at 24 weeks compared with the MITT group (Halpin et al., 2021b). Furthermore, only a retrospective cohort study in Spain showed that SITT was related to a lower risk of exacerbation and lower medical treatment costs (Alcázar-Navarrete et al., 2022).

At present, research has focused on the real-world treatment status and efficacy of triple therapy for COPD mainly in European and American countries. China is a developing Asian country with a lower proportion of people who are over the age of 40 with secondary and tertiary education (including high school and vocational education), and the population, in general, has lower health insurance coverage than in European countries and the United States. These factors may influence the inhalation use and economic burden of SITT and MITT (López-Campos et al., 2019). In November 2019, SITT was listed and put into clinical use for COPD treatment in attending hospitals. There is a lack of real-world studies about the treatment status and therapeutic effects of SITT and MITT in COPD in the Chinese population. This study prospectively aims to compare the treatment persistence, adherence, and risk of exacerbation and mortality in patients with COPD in SITT *versus* MITT groups in the Chinese population.

## Methods

### Study design and subjects

This was a multicenter, prospective observational study. All subjects were outpatients diagnosed with COPD at their first hospital visit from 1 January 2020 to 31 November 2021 in one of ten hospitals in Hunan and Guangxi provinces in China (the Second Xiangya Hospital of Central South University, the Zhuzhou Central Hospital, the Hunan Prevention and Treatment Institute for Occupational Diseases, the First Affiliated Hospital of Shaoyang University, the Eighth Hospital in Changsha, Longshan Hospital of Traditional Chinese Medicine, the Second People’s Hospital of Guilin in Guangxi, Traditional Chinese Medicine Hospital of Changde, the First People’s Hospital of Huaihua City, and Hengyang Central Hospital in Hunan). Patients who met the diagnostic criteria for COPD as defined by the 2020 Global Initiative for Chronic Obstructive Lung Disease (GOLD) recommendations—where spirometry with a ratio of the forced expiratory volume in 1 s to the forced vital capacity (FEV1/FVC) is lower than 0.70 after bronchodilator administration (GBD Chronic Respiratory Disease Collaborators, 2020)—who were more than 40 years old, and were treated with single-inhaler or multiple-inhaler triple therapy were included. The study excluded patients with asthma, bronchiectasis, pneumonia, lung cancer, or severe cardiovascular, kidney, or liver disease.

This study was conducted in accordance with the Declaration of Helsinki and approved by the Ethics Committee of the Second Xiangya Hospital of Central South University. All patients provided written informed consent. The Chinese Clinical Trial Registry Registration number is ChiCTR-POC-17010431.

### Data collection

All patients agreed to three visits. Demographic and clinical characteristics collected at the baseline visit included age, sex, body mass index (BMI), education degree, smoking history, biofuel and occupational exposure history, CAT, Clinical COPD Questionnaire (CCQ), mMRC, pulmonary function, and triple therapy drugs (ICS/LABA/LAMA) including SITT (budesonide/glycopyrronium bromide/formoterol fumarate or fluticasone furoate/umeclidinium bromide/vilanterol) and MITT with two inhalers and exacerbations (moderate-to-severe) in the past year. All patients received inhalation training at the patient health management office after receiving an inhaler on their first visit. Subsequent data on exacerbation, all-cause death, and treatment persistence and adherence were collected at 6- and 12-month follow-ups.

### Study outcome

Study outcomes include adherence, persistence, exacerbation, and all-cause mortality during the 1-year follow-up in the SITT and MITT cohorts of COPD. Adherence was assessed as the proportion of adherent patients, defined as patients with a proportion of days covered (PDC) ≥0.8. PDC is calculated as the total number of days of medication provided divided by the total time of treatment with triple therapy (ICS, LAMA, and LABA). Patients with PDC ≥0.8 were defined as showing good adherence, and the proportion of patients with PDC ≥0.8 at 6 and 12 months was calculated (Mannino et al., 2022). Non-persistence (discontinuation) was defined as >60 days without using SITT or MITT, otherwise defined as persistence. The persistence rate is the proportion of patients who obtain persistence at 6 or 12 months (Alcázar-Navarrete et al., 2022; Mannino et al., 2022). The study also recorded the incidence of moderate-to-severe exacerbation during the 1-year follow-up period. “Moderate” was defined as the exacerbation of respiratory symptoms requiring treatment with oral corticosteroids and/or antibiotics; “severe” was defined as exacerbation requiring emergency room admission or hospitalization during the visit. Frequent exacerbation was also defined as at least two exacerbations during the follow-up period.

### Statistical analysis

SPSS 26.0 (IBM, Armonk, NY, USA) was used to statistically analyze the data. Continuous variables were expressed as the mean ± standard deviation or median and interquartile range, as appropriate. Continuous variables were tested using Student’s t-test; otherwise, non-parametric tests were used for non-normal information. Categorical variables were analyzed using the chi-squared test. A multivariate Cox regression analysis was performed to identify the association between triple therapy and treatment persistence or exacerbation during the 1-year follow-up by including variables that were significant (*p* < 0.05) in the univariate analysis. Propensity score matching (PSM) was conducted using the R 2.15.3 package (http://www.R-project.org). Odds ratio (OR) and 95% confidence intervals (CIs) were calculated using logistic regression. For all data analyses, a *p*-value of <0.05 was considered statistically significant.

## Results

### Baseline characteristics of patients with SITT and MITT

**FIGURE 1:**
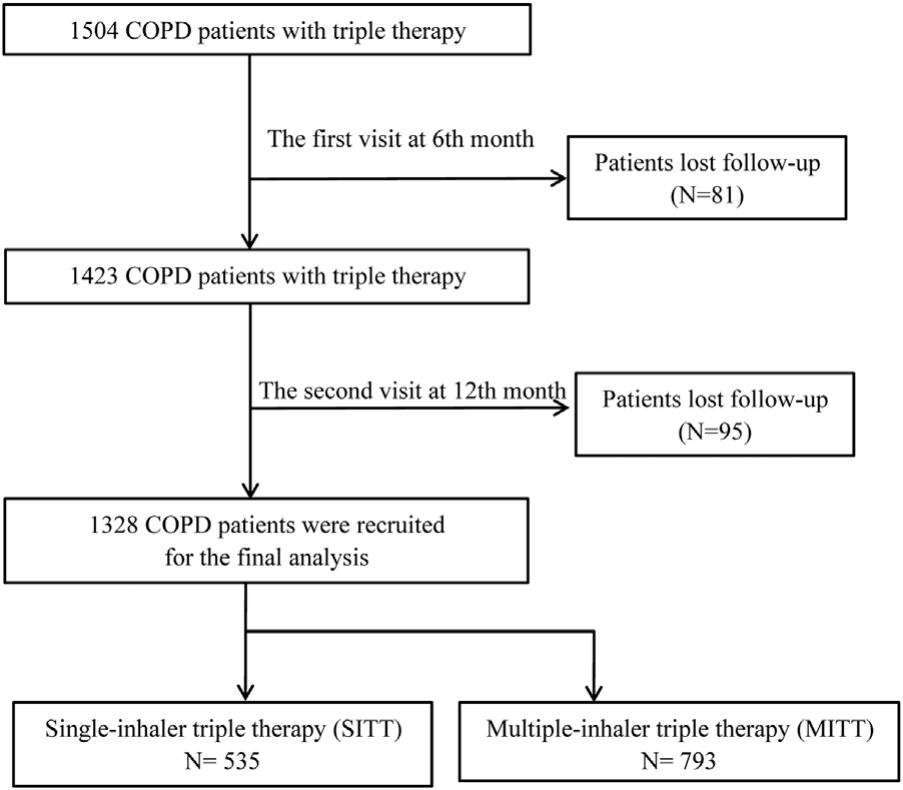
Flow diagram of the study’s inclusion. COPD, chronic obstructive pulmonary disease, MITT, multiple triple-inhaler therapy; SITT, single triple-inhaler therapy.

A total of 1,504 patients with COPD who were treated with triple therapy were initially enrolled. At the 6-month visit, 81 patients were excluded from the study due to loss of contact. At the 12-month follow-up, 95 patients dropped out due to loss of contact. Subsequently, 1,328 patients were recruited for the final analysis, including 535 (40.3%) patients treated with SITT and 793 (59.7%) patients treated with MITT (Figure 1). The baseline characteristics of the study population are summarized in Table 1. The mean age of patients was 64.9 ± 9.2 years; 87.5% of patients were men. The mean CAT and CCQ scores were 15.2 ± 7.1 and 21.9 ± 8.2, respectively, and most patients (76.3%) had an mMRC >1. The all-study patients were divided into GOLD A (8,2%), B (41.9%), C (3.2%), and D 6 (46.8%) groups. In addition, the median (IQR) FEV1% was 54.4 (31.2), and 56.0% of patients had a previous exacerbation history. The baseline characteristics had no significant difference between the study population and patients who were lost to follow-up (Supplementary Table S1).

**TABLE 1:** Baseline characteristics of the study population.

Variable	Total	Single-inhaler triple therapy (SITT)	Multiple-inhaler triple therapy (MITT)	*p*-value
	N = 1328	N = 535	N = 793	
Age (years), mean (SD)	64.9 (9.2)	65.3 (9.2)	64.6 (9.2)	0.146
Sex, n (%)				0.183
Male	1162 (87.5)	476 (89.0)	686 (86.5)	
Female	166 (12.5)	59 (11.0)	107 (13.5)	
Education, n (%)				0.327
Primary school	581 (43.8)	249 (46.5)	332 (41.9)	
Junior high school	477 (36.0)	192 (35.6)	285 (35.9)	
High school	200 (15.0)	69 (12.9)	131 (16.5)	
University	70 (5.2)	25 (4.7)	45 (5.5)	
BMI (kg/m^2^), mean (SD)	23.2 (3.1)	22.6 (3.2)	23.4 (3.4)	0.106
Marriage status, n (%)				0.196
Married	1253 (93.4)	498 (92.2)	746 (94.0)	
Unmarried	75 (6.6)	31 (17.8)	44 (6.0)	
Smoking status, n (%)				0.392
Current smoker	519 (39.1)	215 (44.4)	304 (38.4)	
Ex-smoker	556 (41.9)	228 (42.6)	328 (41.4)	
Non-smoker	252 (19.0)	91 (17.0)	161 (20.3)	
Biofuel exposure, n (%)				0.009
Yes	495 (37.3)	222 (41.5)	273 (34.4)	
No	833 (63.7)	313 (58.5)	520 (65.6)	
Occupational exposure, n (%)				0.203
Yes	546 (41.1)	231 (43.2)	315 (49.7)	
No	782 (58.9)	304 (56.8)	478 (60.3)	
CAT, mean (SD)	15.2 (7.1)	16.2 (6.8)	14.6 (7.1)	< 0.001
mMRC, n (%)				0.013
0–1	273 (23.7)	111 (20.7)	212 (26.7)	
2–4	878 (76.3)	424 (79.3)	581 (73.3)	
CCQ, mean (SD)	21.9 ± 8.2	22.5 ± 8.5	21.5 ± 8.0	0.031
GOLD group				0.000
A	109 (8.2)	31 (5.8)	78 (9.8)	
B	556 (41.9)	200 (37.4)	356 (44.9)	
C	42 (3.2)	13 (2.4)	29 (3.7)	
D	621 (46.8)	291 (54.4)	330 (41.6)	
FEV1L, median (IQR)	1.1 (0.72)	1.1 (0.6)	1.2 (0.7)	0.011
FEV1 (% predicted), mean (SD)	48.6 (17.1)	46.1 (16.0)	50.1 (17.4)	< 0.001
FEV1/FVC, mean ± SD	45.0 (12.6)	43.2 (11.5)	46.0 (12.5)	0.001
Exacerbations in the past year, median (IQR)	01)	01)	01)	0.117
Exacerbations, n (%)				< 0.001
Yes	744 (56.0)	338 (63.2)	406 (51.2)	
No	584 (44.0)	197 (36.8)	387 (48.8)	

**Abbreviations:** BMI, body mass index; COPD, chronic obstructive pulmonary disease; CAT, COPD assessment test; CCQ, clinical COPD questionnaire; FEV1, forced expiratory volume in 1 second; FVC, forced vital capacity; GOLD, Global Initiative for Chronic Obstructive Lung Disease; ICSs, inhaled corticosteroids; IQR, interquartile range; LABA, long-acting β-2-agonist; LAMA, long-acting muscarinic antagonist; mMRC, modified Medical Research Council dyspnoea scale; MITT, multiple triple-inhaler therapy; SITT, single triple-inhaler therapy.

There were no significant differences between the SITT and MITT groups for age, sex, education level, BMI, smoking status, and the proportion of patients with occupational exposure history. However, compared to patients treated with MITT, patients treated with SITT had a higher proportion of biofuel exposure history, a higher mean CAT score (16.2 ± 6.8 vs. 14.6 ± 7.1, *p* < 0.001), higher mean CCQ score (22.5 ± 8.5 vs. 21.5 ± 8.0, *p* = 0.031), and more patients had mMRC >1 [424 (79.3%) vs. 581 (73.3), *p* = 0.013]. In addition, the mean FEV1 (%predicted) [46.1 (16.0) vs. 50.1 (17.4), *p* < 0.001] and FEV1/FVC [424 (79.3%) vs. 581 (73.3), *p* = 0.001] were both lower in the SITT than in the MITT cohort. Moreover, the number of exacerbations during the previous year showed no statistical differences between the two cohorts, but the proportion of patients with ≥1 exacerbation was higher in the SITT than in the MITT group [(338 (63.2%) vs. 406 (51.2%), *p* < 0.001] (Table 1).

### Comparison of treatment persistence and adherence in patients with SITT and MITT

**FIGURE 2:**
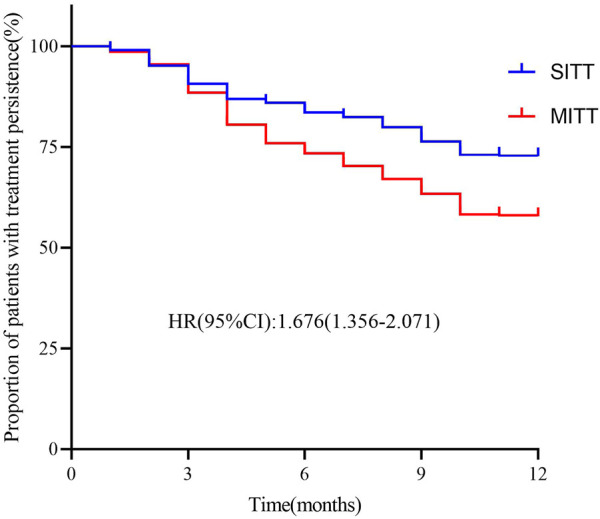
Kaplan–Meier graphs for treatment persistence in SITT and MITT. Adjusted for covariates: age, sex, body mass index, smoking status, forced expiratory volume in 1 s (FEV1), CAT, mMRC, CCQ, and previous exacerbation MITT, multiple triple-inhaler therapy; SITT, single triple-inhaler therapy.

As shown in Table 2, the treatment persistence rate of the study population was 82.5% at 6 months and 63.6% at 12 months; mean treatment persistence days was 287.1 (105.5) during the 12-month visit. The proportion of patients with good medication adherence with PDC ≥0.8 was 82.7% at the 6-month visit and 69.8% at the 12-month visit. In addition, treatment persistence rates in the SITT *versus* the MITT cohorts were higher at both 6 months (80.6% vs. 76.7%; *p* = 0.008) and 12 months (72.5% vs. 57.6%; *p* < 0.001). The mean treatment persistence days in the SITT and MITT groups was 304.6 (97.5) and 275.3 (109.5) days, respectively (*p* < 0.001). Furthermore, the SITT cohort also had a higher proportion of patients with PDC ≥0.8 at both 6 months (86.5% vs. 80.1%; *p* = 0.008) and 12 months (76.8% vs. 65.5%; *p* < 0.001). After adjusting for age, sex, body mass index, smoking status, forced expiratory volume in 1 s (FEV1), CAT, mMRC, CCQ, and previous exacerbation, patients treated with SITT had higher persistence than MITT patients during the 12-month follow-up [hazard ratio (HR): 1.676; 95% CI: 1.356-2.071; *p* < 0.001] in multivariate Cox analysis (Figure 2).

**TABLE 2:** Treatment persistence and adherence during 12-month follow-up.

Variable	Total	Single-inhaler triple therapy (SITT)	Multiple-inhaler triple therapy (MITT)	*p*-value
	N = 1328	N = 535	N = 793	
Persistence, n (%)				
6 months	1096 (82.5)	463 (86.5)	633 (79.8)	0.006
12 months	845 (63.6)	388 (72.5)	457 (57.6)	< 0.001
Treatment persistence days, mean (SD)	287.1 (105.5)	304.6 (97.5)	275.3 (109.5)	< 0.001
PDC>0.8, n (%)				
6 months	1098 (82.7)	463 (86.5)	635 (80.1)	0.008
12 months	927 (69.8)	411 (76.8)	516 (65.1)	< 0.001
Persistence		Cox proportional hazards model**		
HR (95% CI)		1.676 (1.356-2.071)		< 0.001

**Abbreviations:** PDC, proportion of days covered.

### Risk of exacerbation and mortality in patients with SITT and MITT

During the 12-month follow-up, the median number of exacerbations in the entire study population was 01. In addition, 801 (60.3%) patients had at least one exacerbation, 333 (25.1%) had severe exacerbations, and 170 (12.8%) had frequent exacerbations during the 12-month visit. Moreover, patients in the SITT group had a significantly lower median number of exacerbations (*p* = 0.016), a lower proportion of patients with ≥1 exacerbation (33.5% vs. 43.9%; *p* < 0.001), and a lower proportion with severe but frequent exacerbation (20.0% vs. 28.5%; *p* < 0.001) (Table 3). In addition, the SITT cohort had decreased incidence of all-cause mortality (2.4% vs. 4.7%). After using PSM in age, CAT, mMRC, CCQ, FEV1%, previous exacerbation history, and persistence—which was different between the two groups at baseline—there were 494 patients with SITT and 494 with MITT (Supplementary Table S2). In the multivariate Cox analysis, it was evident that SITT may reduce the risk of future moderate-to-severe exacerbation [HR: 0.729 (0.593–0.898), *p* = 0.003] and future severe exacerbation [HR: 0.675 (0.515–0.875), *p* = 0.003], as well as decreased risk of mortality [HR: 0.475 (0.237-0.952), *p* = 0.036] compared with MITT. It was also indicated that persistence with inhalation therapy may reduce the risk of moderate-to-severe exacerbation [HR: 0.401 (0.325–0.495), *p* < 0.001], severe exacerbation [HR: 0.404 (0.309–0.528), *p* < 0.001], and all -cause mortality [HR: 0.405 (0.205–0.800), *p* = 0.009] frequent exacerbation [HR: 0.433 (0.305–0.615), *p* < 0.001] during the 12-month follow-up (Table 4).

**TABLE 3:** Exacerbation rates and all-cause mortality during 12-month follow-up.

Variable	Total	Single-inhaler triple therapy (SITT)	Multiple-inhaler triple therapy (MITT)	*p*-value
	N = 1328	N = 535	N = 793	
Exacerbations in 1 year, mean (SD)	0(1)	0(1)	0(1)	0.017
Exacerbation in 1 year, n (%)				
0	527 (39.7)	356 (66.5)	445 (56.1)	< 0.001
≥1	801 (60.3)	179 (33.5)	348 (43.9)	
Severe exacerbation in 1 year, n (%)				< 0.001
Yes	333 (25.1)	107 (20.0)	226 (28.5)	< 0.001
No	995 (74.9)	428 (80.0)	567 (71.5)	
Frequent exacerbation in 1 year, n (%)				0.452
Yes	170 (12.8)	64 (12.0)	106 (13.4)	
No	1158 (87.2)	471 (88.0)	687 (86.7)	
Mortality, n (%)	50 (3.8)	13 (2.4)	37 (4.7)	0.036

**TABLE 4:** Risk of different inhaler triple therapy and treatment persistence for future exacerbation and mortality during 1-year follow-up using Cox regression after PSM.

	Moderate-to-severe exacerbation		Severe exacerbation		Frequent exacerbation		Mortality	
	HR (95CI%)	*p-*value	HR (95CI%)	*p-*value	HR (95CI%)	*p-*value	HR (95CI%)	*p-*value
SITT (n = 494) *versus* MITT (n = 494)	0.729 (0.593-0.898)	0.003	0.675 (0.515-0.875)	0.003	0.774 (0.548-1.093)	0.146	0.475 (0.237-0.952)	0.036
Persistence (yes *versus* no)	0.401 (0.325-0.495)	< 0.001	0.404 (0.309-0.528)	< 0.001	0.433 (0.305-0.615)	< 0.001	0.405 (0.205-0.800)	0.009

**Notes:** PSM (age, CAT, mMRC, CCQ, FEV1%, previous exacerbation history, and persistence), age, sex, CAT score, mMRC score, CCQ score, FEV1%, exacerbation in the past year, SITT or MITT therapy, and adherence were included as the variables in the multivariate Cox analysis.

**Abbreviations:** MITT, multiple triple-inhaler therapy; SITT, single triple-inhaler therapy.

### Exacerbations and mortality during follow-up according to treatment persistence

**FIGURE 3:**
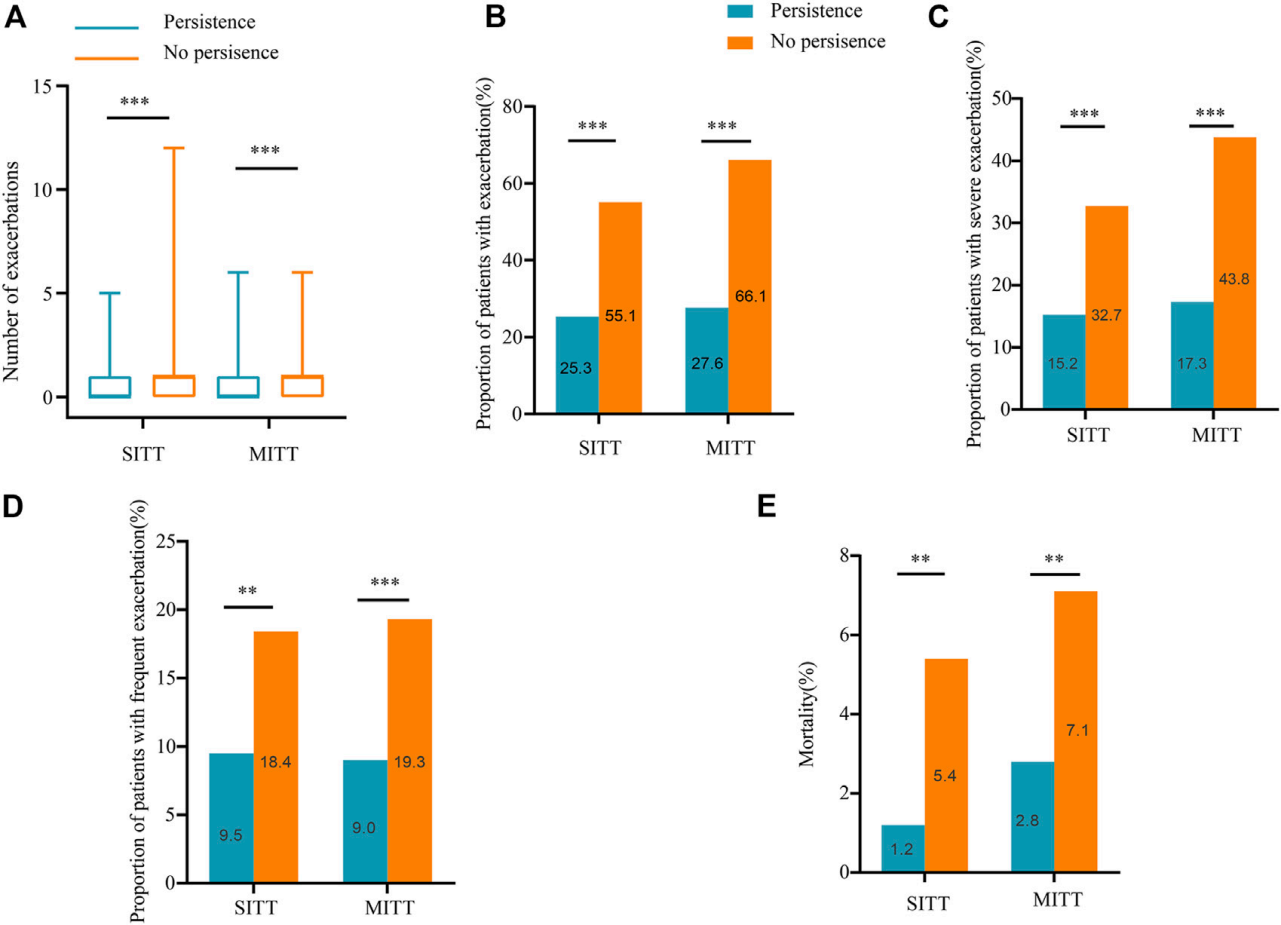
Exacerbations and mortality during follow-up according to treatment persistence. **(A)** Number of exacerbations during 1-year follow-up in patients treated with SITT and MITT according to treatment persistence; **(B)** proportion of patients with exacerbation during 1-year follow-up in patients treated with SITT and MITT according to treatment persistence; **(C)** proportion of patients with severe exacerbation during 1-year follow-up in patients treated with SITT and MITT according to treatment persistence; **(D)** proportion of patients with frequent exacerbation during 1-year follow-up in patients treated with SITT and MITT according to treatment persistence; **(E)** mortality of patients during 1-year follow-up in patients treated with SITT and MITT according to treatment persistence. MITT, multiple triple-inhaler therapy; SITT, single triple-inhaler therapy.

In both the SITT and MITT groups, persistent patients had lower exacerbation rates than non-persistent patients. In the SITT cohort, the median number of exacerbations in persistent *versus* non-persistent patients was [01) *versus* 11)], respectively (*p* < 0.001), and in the MITT cohort was [01) vs. 11) (*p* < 0.001)], respectively. The proportion of patients with exacerbation in the SITT group for persistence and non-persistence was 25.3% vs. 55.1% (*p* < 0.001), respectively; and in the MITT group was 27.6% vs. 66.1% (*p* < 0.001), respectively. Moreover, persistent patients in the SITT and MITT groups both had a lower incidence of severe exacerbation [15.2% vs. 32.7%, *p* < 0.001 and 17.3 vs. 43.8, *p* < 0.001] and frequent exacerbation [9.5% vs. 18.4%, *p* = 0.005 and 9.0 vs. 19.3, *p* < 0.001] than non-persistent patients during the 1-year follow-up (Figure 3). Persistent patients in the SITT and MITT groups also both had a lower incidence of death [1.2% vs. 5.4%, *p* = 0.005 and 2.8 vs. 7.1, *p* = 0.004] than the non-persistent.

## Discussion

Based on data from multiple centers in China, this real-world and prospective observational study demonstrated that patients with SITT had increased treatment persistence and adherence compared to patients with MITT in COPD. SITT reduced the risk of moderate-to-severe exacerbation, severe exacerbation, and mortality during the 1-year follow-up, compared with the MITT therapy.

This study found that patients in the SITT group had a significantly lower median number of exacerbations, a lower proportion of patients with ≥1 exacerbation, and severe but frequent exacerbations during the 1-year follow-up in COPD. This is in accordance with an observational study in Spain which found that patients with COPD in the SITT cohort had a lower median number of exacerbations and lower incidence of moderate-to-severe future exacerbation and all-cause mortality than the MITT cohort during the 12-month follow-up (Alcázar-Navarrete et al., 2022). Furthermore, after adjusting for differences in persistence and other variables in multivariate Cox analysis, the study showed that SITT led to a risk reduction in moderate-to-severe and severe exacerbation during the follow-up, compared with the MITT group. However, previous studies have shown that SITT can enhance symptom improvement in a European 24-week multicenter, randomized, open-label, phase IV effectiveness study (INTREPID) (Halpin et al., 2021b) and reduce the risk of future exacerbation and mortality, but this was not adjusted for differences in treatment persistence (Alcázar-Navarrete et al., 2022). The present study was the first to show that SITT could still decrease the risk of exacerbations and mortality over MITT after adjusting for differences in persistence. This will provide the basis for the clinical selection of therapeutic devices for triple therapy.

Our results also showed that persistence at 12 months for the entire study population was 63.6% and that COPD patients in the SITT group had a higher persistence rate than those in the MITT group. This was in keeping with the published results in which the persistence at 12 months for all patients was 62.7%, and a higher persistence rate was also found in the SITT than in the MITT cohort (Alcázar-Navarrete et al., 2022). These two studies both use a treatment gap of no more than 60 days to define treatment persistence for COPD. Using an allowable treatment gap of no more than 30 days, an analysis of a US study found that 86% of patients discontinued treatment at 12 months (Halpin et al., 2021b). The study also reported that the PDC ≥0.8 of all study participants was 69.8% and that patients in the SITT group had a higher proportion of PDC ≥0.8 than those in the MITT group. Previous studies also indicate that the SITT group had better adherence than the MITT group, but the rate of patients with good adherence for COPD is lower than that of the present study, with a range of 15%–50% (Bogart et al., 2019; Zucchelli et al., 2020; Mannino et al., 2022). This was possibly due to the heterogeneity of the study population and the difference in study time, as well as the fact that most of our study population had received COPD disease awareness education and inhalation training. Moreover, persistence was associated with improved moderate-to-severe, severe, and frequent exacerbation rates, as well as a decreased probability of death compared with non-persistence in both the SITT and MITT groups. It is also consistent with a previous study in Spain (Alcázar-Navarrete et al., 2022). In addition, our study first discovered that persistence to inhalation therapy was an independent risk factor of future moderate-to-severe exacerbation, severe exacerbation, and frequent exacerbation during follow-up.

This real-world study indicated that the SITT group had a higher proportion of biofuel exposure history, higher mean CAT score, higher mean CCQ score, and more patients with mMRC >1. In addition, the mean FEV1 (% predicted) and FEV1/FVC were both lower in the SITT than in the MITT cohort. The proportion of patients with ≥1 exacerbation was higher in the SITT cohort at baseline. This part of the results is different from previous studies in which the baseline characteristics were mostly similar between the two cohorts (Halpin et al., 2021b; Alcázar-Navarrete et al., 2022). Therefore, we matched the different baseline characteristics of the two cohorts using PSM and then used Cox regression analysis to analyze the relation between SITT or MITT and the risk of exacerbation.

The study has a few potential limitations. First, inhaler use by patients and the inhalation technique could not be ascertained. The second is that the diagnosis of an exacerbation of COPD was based on drug claims (moderate exacerbation) and hospital admission or emergency visits (severe exacerbation).

## Conclusion

Patients with COPD treated with SITT had improved treatment persistence and adherence, as well as a risk reduction of moderate-to-severe exacerbation, severe exacerbation, and all-cause mortality compared to patients with MITT in the Chinese population.

## Data Availability

The original contributions presented in the study are included in the article/Supplementary Material; further inquiries can be directed to the corresponding author.

## Abbreviations

BMI, body mass index; COPD, chronic obstructive pulmonary disease; CAT, COPD assessment test; FEV1, forced expiratory volume in one second; FVC, forced vital capacity; GOLD, Global Initiative for Chronic Obstructive Lung Disease; ICS, inhaled corticosteroids; IQR, interquartile range; LABA, long-acting β-2-agonist; LAMA, long-acting muscarinic antagonist; mMRC, modified Medical Research Council; MITT, multiple triple-inhaler therapy; PDC, proportion of days covered; ROC, receiver operating characteristic; SITT, single triple-inhaler therapy.
