# Supplementary material for: Comparison of treatment persistence, adherence, and risk of exacerbation in patients with COPD treated with single-inhaler versus multiple-inhaler triple therapy: A prospective observational study in China
Source: Front Pharmacol. 2023 Mar 21;14:1147985. doi: 10.3389/fphar.2023.1147985 (PMC10072324; doi:10.3389/fphar.2023.1147985)
Supplement: Supplementary file 1 [file DataSheet1.pdf]

Supplement Table 1: Baseline characteristics of the study population and missing data

Variables	Study population N=1328	Missing data N=176	P-value
Age (years), Mean (SD)	64.9 (9.2)	64.8 (9.1)	0.746
Sex,n(%)			0.183
Male	1162(87.5)	158(89.8)	
Female	166(12.5)	18(10.2)	
Education,n(%)			0.298
Junior high school and less	1058(79.8)	144(81.6)	
High school and above	270(20.2)	32(18.4)	
BMI (kg/m^2), Mean (SD)	23.2(3.1)	22.9 (3.4)	0.406
Marry status,n(%)			
married	1253(93.4)	162(91.8)	0.199
unmarried	75(6.6)	14(8.2)	
Smoking state,n(%)			0.422
Current smoker	519(39.1)	78(44.1)	

Ex-smoker	556(41.9)	71(40.6)	
Non-smoker	252(19.0)	27(15.3)	
Biofuel exposure,n(%)			**0.005**
Yes	495(37.3)	84(47.7)	
No	833(63.7)	92(52.3)	
Occupational exposure,n(%)			0.113
Yes	546(41.1)	78(44.1)	
No	782(58.9)	98(55.9)	
CAT, Mean (SD)	15.2 (7.1)	14.5 (6.9）	0.091
mMRC,n(%)			**0.043**
0-1	273(23.7)	47(26.8)	
2-4	878(76.3)	129(73.2)	
CCQ, Mean (SD)	21.9 ± 8.2	21.5 ± 7.6	0.375
FEV1(L), Median (IQR)	1.1(0.72)	1.1(0.69)	0.211
FEV1 (% predicted), Mean (SD)	48.6(17.1)	48.3(16.2)	0.345

FEV1/FVC, Mean (SD)	45.0(12.6)	45.5(11.9)	0.278
Exacerbations,n(%)			0.161
Yes	744(56.0)	94(53.1)	
No	584(44.0)	82(36.8)	

Abbreviations: BMI, Body Mass Index; COPD, Chronic Obstructive Pulmonary Diseas; CAT, COPD Assessment Test; CCQ, Clinical COPD Questionnaire; FEV1, Forced Expiratory Volume in one second; FVC, Forced Vital Capacity; GOLD, Global Initiative for Chronic Obstructive Lung Disease. ICS, inhaled corticosteroids; IQR, interquartile range; LABA, long-acting β-2-agonist; LAMA, long-acting muscarinic antagonist; mMRC, modified medical research council dyspnea scale; MITT, multiple triple-inhaler therapy; SITT, Single triple-inhaler therapy

Supplement Table 2: The clinical characteristics of COPDpatients with SITT therapy and MITT after propensity score matching

Variables	Total N=992	SITT N=494	MITT N=494	P-value
Age (years), Mean (SD)	65.0(9.1)	65.1 (9.3)	65.0 (9.0)	0.920
Sex,n(%)				0.173
Male	868(87.9)	441(89.3)	427(86.4)	

BMI (kg/m^2), Mean (SD)	23.0(3.1)	22.9(3.0)	23.3 (3.3)	0.406
Marry status,n(%)				
married	914(92.1)	461(93.3)	453(91.7)	0.200
unmarried	78(7.9)	33(6.7)	41(8.3)	
Smoking state,n(%)				0.515
Current smoker	383(38.8)	200(40.5)	183(40.0)	
Ex-smoker	423(42.8)	211(42.7)	212(42.9)	
Non-smoker	182(18.4)	83(16.8)	99(20.0)	
Biofuel exposure,n(%)				0.362
Yes	390(39.5)	202(40.9)	188(38.1)	
No	598(60.5)	292(59.1)	306(61.9)	
CAT, Mean (SD)	15.8(7.1)	15.9 (7.0)	15.8 (7.2）	0.815
mMRC,n(%)				0.443
0-1	252(25.4)	125(25.3)	127(25.7)	
2-4	736(74.6)	369(74.7)	367(74.3)	
CCQ, Mean (SD)	22.5(7.9)	22.5 (8.0)	22.4 (7.9)	0.852

FEV1 (% predicted Mean (SD)	46.9(17.1)	46.4(17.0)	47.5(17.0)	0.212
FEV1/FVC, Mean (SD)	44.0(12.0)	43.7(11.6)	44.3(12.3)	0.506
Exacerbations,n(%)				0.696
Yes	596(60.3)	301(60.9)	295(59.7)	
No	392(39.7)	193(39.1)	199(40.3)	
Persistent,n(%)				1.000
Yes	698(70.6)	349(70.6)	349(70.6)	
No	290(29.4)	145(29.4)	145(29.4)	

Abbreviations: BMI, Body Mass Index; COPD, Chronic Obstructive Pulmonary Diseas; CAT, COPD Assessment Test; CCQ, Clinical COPD Questionnaire; FEV1, Forced Expiratory Volume in one second; FVC, Forced Vital Capacity; GOLD, Global Initiative for Chronic Obstructive Lung Disease. ICS, inhaled corticosteroids; IQR, interquartile range; LABA, long-acting β-2-agonist; LAMA, long-acting muscarinic antagonist; mMRC, modified medical research council dyspnea scale; MITT, multiple triple-inhaler therapy; SITT, Single triple-inhaler therapy
